# ﻿*Mazusmotuoensis* (Mazaceae), a new species from Xizang, China

**DOI:** 10.3897/phytokeys.235.111092

**Published:** 2023-11-14

**Authors:** Wen-Bin Ju, Xiong Li, Heng-Ning Deng, Meng Li, Xing-Jin He, Xin-Fen Gao, Bo Xu

**Affiliations:** 1 Key Laboratory of Mountain Ecological Restoration and Bioresource Utilization & Ecological Restoration Biodiversity Conservation, Chengdu Institute of Biology, Chinese Academy of Sciences, Chengdu 610041, Sichuan, China Chengdu Institute of Biology, Chinese Academy of Sciences Chengdu China; 2 Key Laboratory of Bio-Resources and Eco-Environment of Ministry of Education, College of Life Sciences, Sichuan University, 610065, Chengdu, Sichuan, China Sichuan University Chengdu China; 3 University of Chinese Academy of Sciences, Beijing 100049, China University of Chinese Academy of Sciences Beijing China; 4 Co-Innovation Center for Sustainable Forestry in Southern China, College of Biology and the Environment, Nanjing Forestry University, Nanjing, 210037, China Nanjing Forestry University Nanjing China

**Keywords:** *Mazus*, molecular phylogenetics, morphology, taxonomy

## Abstract

*Mazusmotuoensis* W.B.Ju, Bo Xu bis & X.F.Gao is a newly described species found in Xizang Autonomous Region, China. Morphologically, this species differs from all the other known *Mazus* species by having erect perennial herb form with a rhizome, presence of multicellular hairs, without basal leaves, opposite arrangement of stem leaves, and corolla lobes with erose-toothed margins. Molecular phylogenetic analysis using nuclear and cpDNA genes suggests that this new species occupies a basal position within *Mazus*. In conclusion, both morphological evidence and molecular phylogenetic analyses support that this species belongs to *Mazus* and represents an as-yet-unreported new species with distinct differences from other species within the genus.

## ﻿Introduction

*Mazus* Loureiro is the largest genus within the family MazaceaeReveal (2011), comprising 38 accepted species (POWO 2022). Most of these species are found in eastern and southeastern Asia, Australia, and New Zealand (Li 1954; Hsieh 2000). The genus is characterized by a distinct two-lipped corolla (3/2-bilabiate), a palate with two longitudinal plaits, and a capsule enclosed in a persistent calyx (Fischer 2004; Deng et al. 2019). In China, there are approximately 31 species and three varieties have been recognized (Hong et al. 1998; Hsieh 2000; Deng et al. 2016; Ying 2019; Xiang et al. 2021; Li et al. 2022), which is the distribution and diversity center of the genus (Hsieh 2000). Originally categorized under Scrophulariaceae through morphological studies (Von Wettstein 1891; Thieret 1954, 1967; Hong et al. 1998), molecular phylogenetic analyses unveiled a robustly supported clade uniting *Mazus* and *Lancea* Hook.f. & Thomson, recognized as the subfamily Mazoideae within Phrymaceae (Beardsley and Olmstead 2002). Nevertheless, subsequent phylogenetic studies confirmed that *Mazus* should be separated from Phrymaceae (Oxelman et al. 2005; Albach et al. 2009; Xia et al. 2009; Schäferhoff et al. 2010), leading to the establishment of a new family called Mazaceae within the Lamiales. The latest phylogenetic studies and morphological evidence indicate that *M.lanceifolius* Hemsly is a distinct species, positioned at the most basal branch within the Mazaceae family, and is the sister genus to the three recognized genera *Dodartia*, *Lancea*, and *Mazus* (Xia et al. 2012; Deng et al. 2019; Xiang et al. 2021).

In 2022, a field survey was conducted in Motuo County, located within the Xizang Autonomous Region of southwest China, the authors discovered an unknown species of Mazaceae in an evergreen broad-leaved forest. Through careful comparison with specimens, related literature, and phylogenetic analysis of Mazaceae, it was concluded that this species represents a new addition to the *Mazus*.

## ﻿Materials and methods

### ﻿Morphological analysis

One population of this new species was rediscovered in Mar 2022 in Xizang Autonomous Region, China. Morphological observations of the new species were conducted using living plants collected from the type locality, as well as type specimens deposited at CDBI. Detailed photographs of morphological features, such as rhizomes, multicellular hairs, stems, leaves, inflorescences, and flowers, were taken using a digital camera and stereoscope. Measurements were carried out on both wild plants and pressed specimens using a ruler and a metric vernier caliper. Digital herbarium images of *Mazus* specimens were sourced from diverse outlets, including JSTOR Global Plants (http://plants.jstor.org/), the Global Biodiversity Information Facility (https://www.gbif.org/zh/), the Chinese Virtual Herbarium (https://www.cvh.ac.cn/), and Europeana (https://www.europeana.eu/en/search). A thorough examination and comparison of these images with the new species ensued. Subsequently, the morphological attributes of the species were meticulously described in accordance with the guidelines provided by the Flora of China (Hong et al. 1998).

### ﻿Assessment of conservation status

In the field, we conducted an estimation of the population size of the new species and evaluated the factors posing threats to its existence. In order to determine the conservation status of the new species, we applied the established criteria as outlined by the International Union for Conservation of Nature (IUCN 2019) Red List.

### ﻿DNA sequencing and outgroup selection

We extracted total DNA from silica gel-dried leaves of the new species using a modified CTAB protocol (Doyle and Doyle 1987). To determine the phylogenetic position of the new species within the *Mazus* genus, we employed two datasets for our analysis. The first dataset consisted of a combined matrix of two cpDNA regions (*rbcL*, *trnL*-*trnF*), while the second dataset was nrITS. The DNA sequences were amplified and sequenced following the methods described by Deng et al. (2019), using the primers specified in their study. Based on previous phylogenies (Deng et al. 2019; Xiang et al. 2021), 20 species with 28 accession of the relatives of *M.motuoensis* were selected as ingroups. Additionally, we chose five species from three different genera as outgroups. The related sequences were obtained from NCBI (https://www.ncbi.nlm.nih.gov/). The GenBank accession numbers for the new species are OQ383430 (trnL-F), OQ383431 (rbcL) and OP720888 (ITS). A comprehensive list of all species included in the phylogenetic analysis, along with their respective accession numbers, can be found in Table 1.

**Table 1.** Information of samples used for phylogenetic inference in this study.

Taxa	rbcL	trnL-F	ITS
*Mazusalpinus* Masamune 1	KX783481	KX783520	MK192641
*Mazusalpinus* Masamune 2	KX783480	KX783519	MK192642
*Mazuscaducifer* Hance 1	KX783477	KX783516	MK192664
*Mazuscaducifer* Hance 2	KX783487	KX783526	MK192659
*Mazuscelsioides* Handel-Mazzetti	KX783486	KX783525	●
*Mazusfruticosus* Bo Li, D.G.Zhang & C.L.Xiang 1	KX783470	KX783509	MK192660
*Mazusfruticosus* Bo Li, D.G.Zhang & C.L.Xiang 2	KX783471	KX783510	MK192649
*Mazusgracilis* Hemsley	FJ172729	FJ172687	FJ172738
*Mazushumilis* Handel-Mazzetti	●	MK266421	MK192667
*Mazusjaponicus* (Thunburg) O. Kuntze	FJ172728	FJ172686	●
Mazusjaponicusvar.delavayi (Bonati) Tsoong	KX783482	KX783521	●
*Mazuslongipes* Bonati	KX783474	KX783513	MK192652
*Mazusmiquelii* Makino 1	KX783475	KX783514	MK192637
*Mazusmiquelii* Makino 2	KX783476	KX783515	MK192655
*Mazusmiquelii* Makino 3	KX783483	KX783522	MK192656
*Mazusmotuoensis* W.B.Ju, Bo Xu bis & X.F.Gao	OQ383431	OQ383430	OP720888
*Mazusnovaezeelandiae* W.R.Barker	KX783469	KX783508	MK192676
*Mazusomeiensis* H. L. Li 1	KX807209	KX807208	MK192636
*Mazusomeiensis* H. L. Li 2	FJ172731	FJ172688	MK192663
*Mazusprocumbens* Hemsley	KX783478	KX783517	MK192647
*Mazuspulchellus* Hemsley	KX783472	KX783511	MK192638
*Mazuspumilio* R.Brown	KX783468	KX783507	MK192671
*Mazuspumilus* (N. L. Burman) Steenis 1	MK266346	KX807206	MH711724
*Mazuspumilus* (N. L. Burman) Steenis 2	HM850162	KX807207	FJ172737
*Mazusreptans* N.E. Brown	HQ384872	AF479004	AF478940
*Mazusspicatus* Vaniot	FJ172730	FJ172689	FJ172740
*Mazussunhangii* D. G. Zhang & T. Deng 1	KX783485	KX783524	●
*Mazussunhangii* D. G. Zhang & T. Deng 2	KX783484	KX783523	●
*Mazusxiuningensis* X. H. Guo & X. L. Liu	MK266349	MK266430	●
**OUTgroup**			
*Puchiumazuslanceifolius* (Hemsly) Bo Li, D. G. Zhang & C. L. Xiang 1	MW373737	MW373741	MW364623
*Puchiumazuslanceifolius* (Hemsly) Bo Li, D. G. Zhang & C. L. Xiang 2	MW373738	MW373742	MW364624
*Dodartiaorientalis* Linnaeus	JQ342984	JQ342981	JQ342980
*Lanceatibetica* J. D. Hooker & Thomson 1	KX783467	KX807205	MK192678
*Lanceatibetica* J. D. Hooker & Thomson 2	MF786661	FJ172685	FJ172736

● refers to a missing sequence.

### ﻿Phylogenetic analysis

The sequence chromatograms were visually inspected on Sequencher 5.2.4 (Gene Codes Corporation) and integrated into a single sequence. All sequences were then aligned with MUSCLE in MEGA 7.0.14 (Kumar et al. 2016) and manually adjusted. Phylogenetic analyses were performed based on the combined cpDNA dataset (*rbcL* and *trnL*-*trnF*) and the nrITS dataset using both the maximum likelihood (ML) and Bayesian inference (BI), respectively. We did not combine the cpDNA and nrITS datasets for analysis because of the different sampling of taxa in the datasets. Settings of parameters during analysis follow those presented in Deng et al. (2019) and Xiang et al. (2021).

## ﻿Results and discussion

### ﻿Morphological analysis

Morphologically, the new species has intermediate characteristics of *Mazus* and *Puchiumazus*. The new species has characteristics such as rhizomes, erect stems, and stem leaves opposite similar to *Puchiumazus*. However, the plant of this new species is covered with multicellular hair and has stems that are not quadrangular and leaf blade elliptic-ovate, which distinguishes it from the only known species, *Puchumazuslancefolius* (Hemsley) Bo Li, D.G.Zhang, and C.L.Xiang (Xiang et al. 2021). There are also perennial herb species with erect stems and opposite cauline leaves in the genus of *Mazus*, such as *M.caducifer*Hance (1882). But the new species has a series of ray characteristics not commonly seen in *Mazus*, including single erect unbranched stems without basal leaves, stem leaves many and opposite, petioles nearly absent, lobes margin erose-toothed. In conclusion, based on the morphological key provided by Hong et al. (1998), the new species is classified morphologically within the *Mazus* and represents an anomalous existence.

### ﻿Phylogenetic analysis

**Figure 1.**
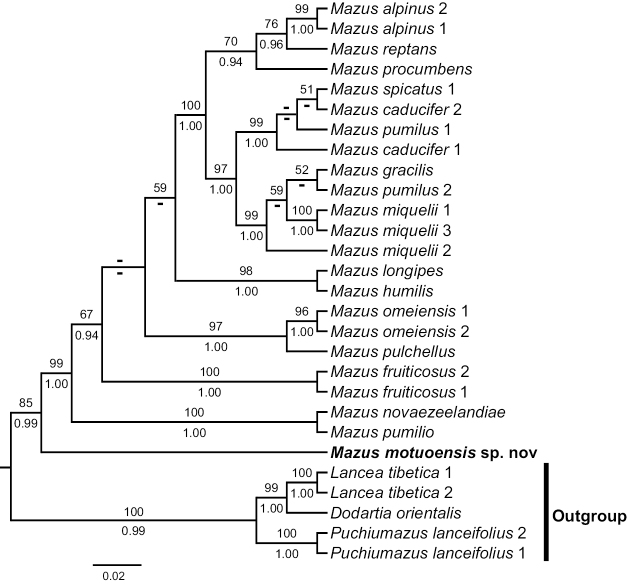
Phylogenetic relationships of *M.motuoensis* and related species inferred from ML and BI analyses based on the nrITS dataset. Numbers on the branches indicate the bootstrap support of the ML and the posterior probability of BI analyses.

**Figure 2.**
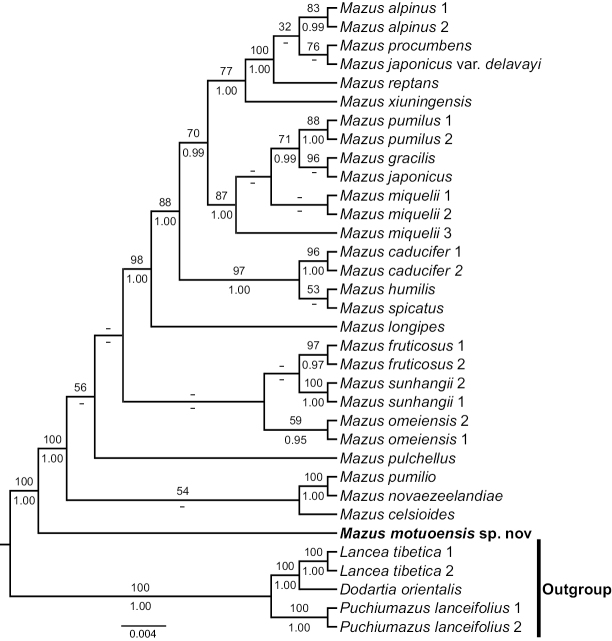
Phylogenetic relationships of *M.motuoensis* and related species inferred from ML and BI analyses based on the combined dataset of *rbcL* and *trnL*-*trnF*. Numbers on the branches indicate the bootstrap support of the ML and the posterior probability of BI analyses.

The phylogenetic tree was generated using a combined cpDNA matrix, consisting of 34 aligned sequences and comprising 2197 characters (*rbcL*: 1318 bp; *trnL*-*trnF*: 879 bp). Additionally, the nrITS matrix included 28 aligned sequences and comprised 703 characters. Due to differences in taxon sampling between the cpDNA and nrITS datasets, they were not combined for analysis. Both maximum likelihood (ML) and Bayesian inference (BI) methods yielded congruent topologies. Therefore, only the results of the ML trees are presented (Figs 1, 2: MLBS: 100%, BIPP: 1.00; all support values follow this order hereafter).

The results of nrITS and cpDNA phylogenies (Figs 1, 2) show that *Dodartia*-*Lancea* formed the basal clade with *Puchiuomazus* clade as sister to *Mazus* (100%, 0.99 in nrITS tree; 100%, 1 in cpDNA tree). The new species was located at the base of the genus *Mazus* with other sampled species of *Mazus* together forming monophyletic clade with strong support (Fig. 1: 85%, 0.99; Fig. 2: 100%, 1). This phylogenetic result is broadly consistent with previous studies (Xiang et al. 2021), indicating that the new species belongs to the *Mazus* representing a unique presence of the genus.

## ﻿Taxonomic treatment

### ﻿ Mazus motuoensis

Taxon classificationPlantaeLamialesMazaceae

W.B.Ju, Bo Xu bis & X.F.Gao sp. nov.

4DD54B86-942B-590A-AFF5-4C86B655823E

urn:lsid:ipni.org:names:77330788-1

Figs 3 , 4 , 5

#### Diagnosis.

The new species is distinguished from congeneric species by its rhizomes, perennial herb covered with multicellular white villus, erect and unbranched stems, having no basal leaves, stem leaves opposite, subsessile, lower lobes margins erose-toothed.

#### Type.

China, Xizang, Motuo County, DeXing town, Nibi Valley, ditch edge in the forest, 29°22'27.98"N, 95°10'0.88"E, alt. 2253 m. 31 Mar 2022, *WenBin JU & XIONG LI, YLZB08519* (holotype: CDBI0279767; isotypes: CDBI0279765, CDBI0279766)

#### Description.

Perennial herbs, 15–25 cm tall, the whole plant is covered with long white soft multicellular hairs. Rhizome white. ***Stems*** erect, unbranched. ***Leaves*** opposite, numerous, petiole inconspicuous to nearly absent; lower leaf blade scalelike and small, obovate-oblong, apex obtuse, middle and upper leaves with leaf blade elliptic to ovate, papery, 0.8–4.0 × 0.4–1.8 cm, adaxially clothed with multicellular hairs, abaxially subglabrous, multicellular hairs on veins, base cuneate, margin serrate, lateral veins 3–5 pairs. ***Racemes*** terminal, ascending to 5 cm long, lax, fewer than 5; pedicels 4–6 mm, glabrous or with a few multicellular hairs; bracts tiny, narrowly lanceolate to linear, glabrous. ***Calyces*** broadly campanulate, ca. 6 mm long, 5–veined, glabrous outside and inside, lobes 5, triangular-lanceolate, as long as tube, apex acute, midrib conspicuous, lateral veins inconspicuous. ***Corolla*** 1.2–1.5 cm long, white, but often purple on upper lobes, glabrous outside and inside apart from clavate hairs on palate; tube 0.3–0.5 cm long, shorter than calyx; limb 2–lipped, upper lip bilobed, slightly upwarp, lobes triangular ovate, apex subacute, sometimes weakly obtuse or retuse; lower lip trilobed, lobes margins erose-toothed, middle lobe usually rounded, smaller than lateral lobes, yellow palate comprising 2 longitudinal elevations extending from point of filament fusion to the base of lower lobes, with erect clavate hairs. ***Stamens*** 4, didynamous, glabrous, inserted at the same level in distal part of tube, inserted at the distal end of the tube at the same level, included; anterior pair longer, curved, appressed to corolla tube, posterior pair spreading; anthers bithecal, locules divergent, apically connivant, positioned adjacent to corolla tube on upper lip; filaments filiform, glabrous. ***Ovary*** ca. 2 mm long, glabrous, ovoid; styles ca. 7 mm long, included, glabrous, exserted beyond anthers, stigma bilobed. Fresh capsule and calyx light green, included by persistent calyx.

**Figure 3.**
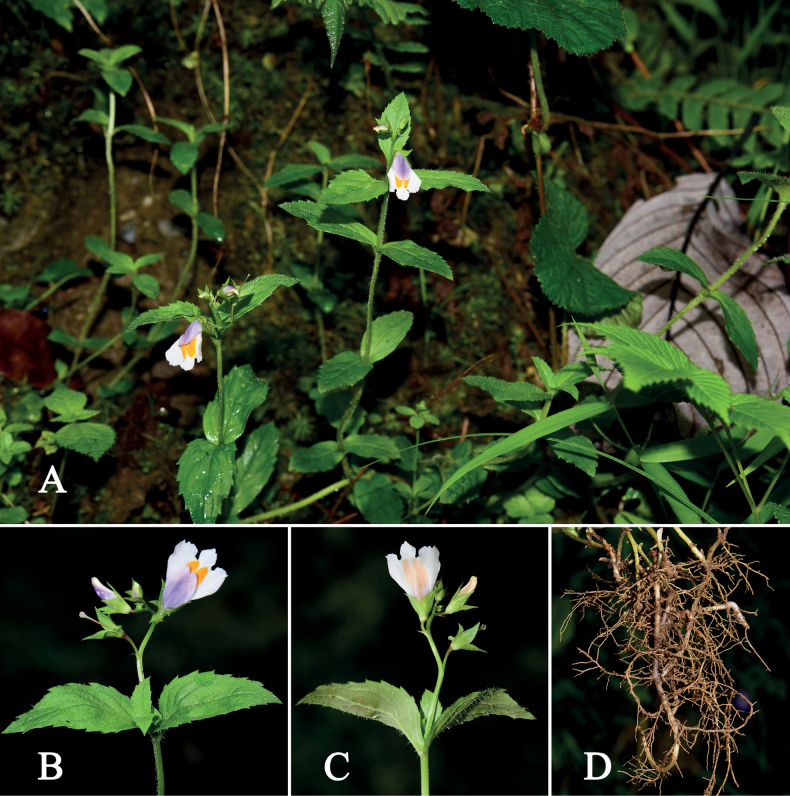
Living images of *M.motuoensis***A** habit **B** inflorescence in frontal view **C** inflorescence in rear view **D** rhizome.

**Figure 4.**
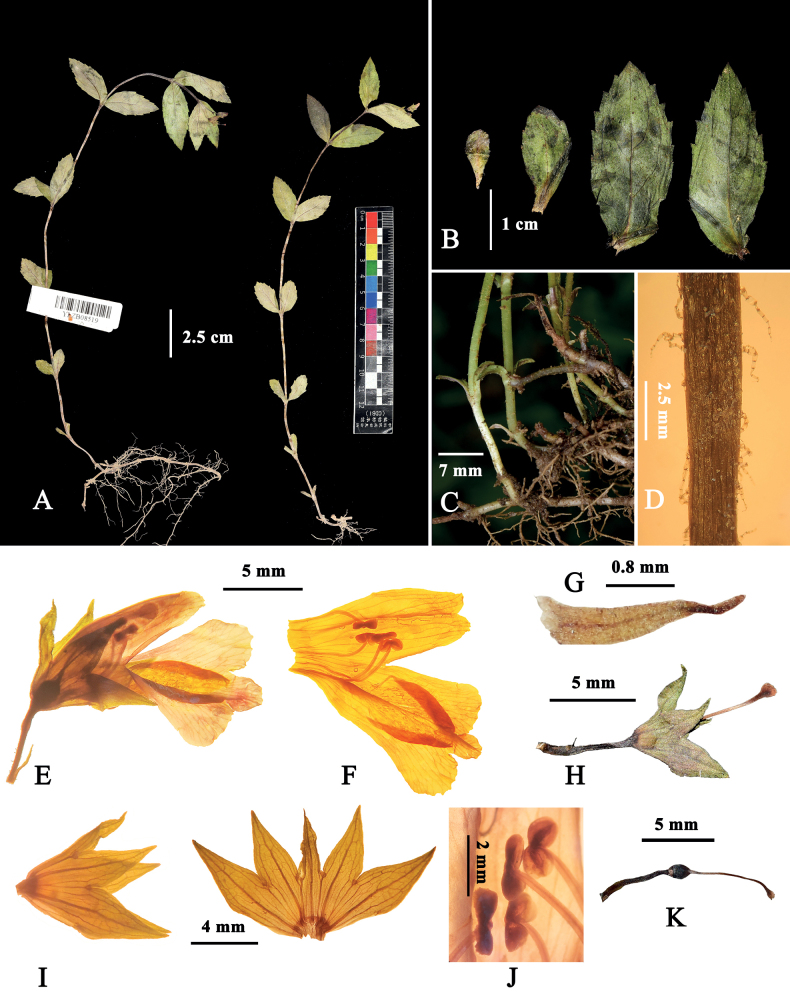
Morphology of *M.motuoensis***A** plant **B** leaves **C** old stems **D** multicellular hairs attached to stem **E** flower **F** unfolded corolla, showing limb upper lip and lower lip **G** bract **H, I** calyx **J** anthers **K** ovary and style.

**Figure 5.**
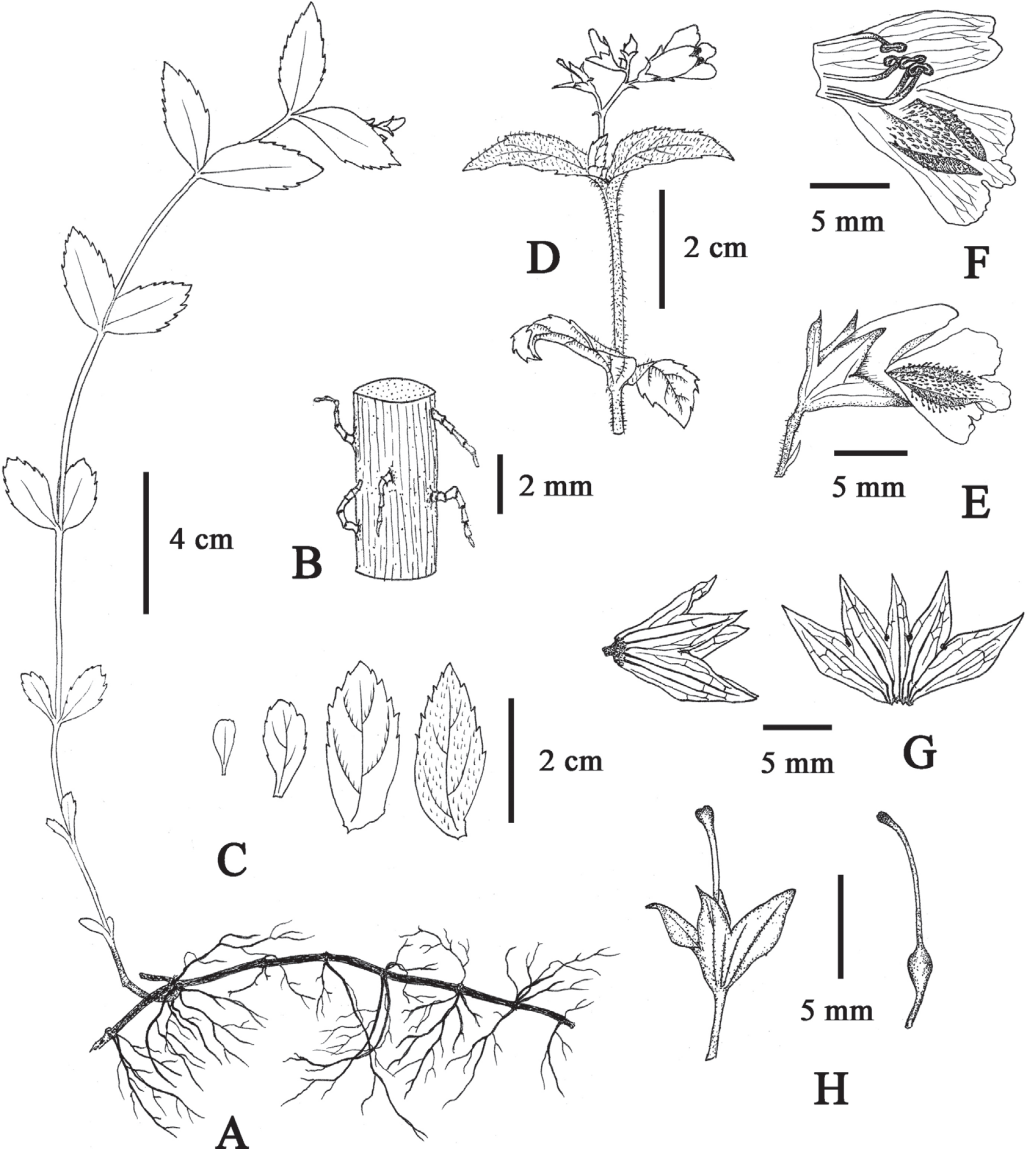
Line drawings of *M.motuoensis***A** whole plant **B** multicellular hairs attached to stem **C** leaves **D** inflorescence **E** flower **F** unfolded corolla **G** calyx **H** ovary and style. Drawn by Mr. Zhen-long Liang.

#### Distribution and habitat.

*Mazusmotuoensis* is currently known from Nibi Valley, Motuo County, Xizang, China. It can be found under evergreen broad-leaved forest at altitudes of 2253 m.

#### Phenology.

Flowering was observed from May to June.

#### Etymology.

The specific epithet “motuoensis” refers to the locality, Motuo County, Xizang, China.

#### Vernacular name.

Simplified Chinese: 墨脱通泉草; Chinese pinyin: Mòtuō Tōngquáncǎo.

#### Conservation status.

Currently, the authors have discovered only one population of *Mazusmotuoensis* from one single locality in Nibi Valley of Motuo County in Xizang Province, China, and ca. 30 individuals from the type locality. Evergreen broad-leaved forests are widely distributed in this area, so we speculate that this new species has a relatively wide distribution range. Due to insufficient field survey, the natural distribution of this species in the wild is not clear. Following the IUCN Red List criteria (2019), we suggest this species placement in the Data Deficient.

## Supplementary Material

XML Treatment for Mazus motuoensis

## ﻿Additional information

### Author ORCIDs

Wen-Bin Ju https://orcid.org/0000-0002-5665-6290

Xiong Li https://orcid.org/0000-0002-1450-8679

Heng-Ning Deng hhttps://orcid.org/0000-0002-0581-0649

Meng Li https://orcid.org/0000-0003-2085-3388

Xing-Jin He https://orcid.org/0000-0003-2064-0112

Xin-Fen Gao https://orcid.org/0000-0002-5703-1639

Bo Xu https://orcid.org/0000-0003-3507-9321

### Data availability

All of the data that support the findings of this study are available in the main text.
